# Evaluation of Borage (*Borago officinalis* L.) Genotypes for Nutraceutical Value Based on Leaves Fatty Acids Composition

**DOI:** 10.3390/foods11010016

**Published:** 2021-12-22

**Authors:** Celia Montaner, Raquel Zufiaurre, María Movila, Cristina Mallor

**Affiliations:** 1 Escuela Politécnica Superior de Huesca, Universidad de Zaragoza, Calle de Cuarte s/n, 22071 Huesca, Spain; zufi@unizar.es (R.Z.); mmovila@unav.es (M.M.); 2 Instituto Agroalimentario de Aragón-IA2, Centro de Investigación y Tecnología Agroalimentaria de Aragón (CITA), Universidad de Zaragoza, 50059 Zaragoza, Spain; 3 Instituto Universitario de Investigación en Ciencias Ambientales de Aragón-IUCA, Universidad de Zaragoza, 50059 Zaragoza, Spain; 4 Centro de Investigación y Tecnología Agroalimentaria de Aragón (CITA), Avda. Montañana, 930, 50059 Zaragoza, Spain

**Keywords:** borage germplasm, vegetable, health, leaf blades, leaf petioles

## Abstract

Borage (*Borago officinalis* L.) is a traditional vegetable grown and consumed in some Spanish regions. The objective of this study was to determine the variability and evolution of fatty acid composition in a borage germplasm collection formed by wild types, breeding lines, commercial varieties, and landraces. Fatty acids were analysed in petioles, the commonly edible part of the leaves, and the leaf blades, the by-product of the borage industry, in two growth stages: at the optimal harvest period (120 days after sowing) and at the end of the harvest period (150 days after sowing). The results showed that for each of the eight fatty acids identified, there were significant differences among the twelve borage genotypes depending on the developmental plant stage at sampling date and the part of the leaf analysed, the interaction effect also being statistically significant. The main polyunsaturated fatty acids identified were: linoleic acid (18:2 n6, LA), α-linolenic acid (18:3 n3, ALA), γ-linolenic acid (18:3 n6, GLA), and stearidonic acid (SDA, 18:4, n-3), account for approximately 70% of polyunsaturated fatty acids. Blue-flowered genotypes differ from white-flowered genotypes by their high content of ALA and SDA, which can be exploited in borage breeding programs. Petioles from young plants present higher n6 fatty acids, while older plants produce a great amount of n3 fatty acids. Besides, the higher content of ALA in the leaf blades gives them a good dietary potential. All these fatty acids, with multiple health benefits, support the nutraceutical interest of borage leaves (both petioles and leaf blades) for human consumption, animal feeding, medicine, and pharmacy.

## 1. Introduction

Healthy eating is one of the most pursued objectives in today’s society and people tend to select food according to its health benefits [1]. According to the Food and Agricultural Organization (FAO) and the World Health Organization (WHO), a healthy diet should include at least 400 g of fruit and vegetables per day, which reduces the risk of non-communicable diseases [2]. Insufficient fruit and vegetable intake is estimated to cause around 31% of ischaemic heart disease and 11% of stroke worldwide [3]. Borage (*Borago officinalis* L.) serves as a nutraceutical plant, making it highly valuable for pleiotropic uses [4].

Nutraceuticals are considered food or part of food, or any substance of both plant and animal origin, which has positive effects on health, playing an important role in maintaining the normal physiological function that keeps humans healthy, including the prevention and/or treatment of diseases. Food sources used as nutraceuticals can be categorized as dietary fiber, prebiotics, probiotics, fatty acids, antioxidant vitamins, polyphenols, and other different types of herbal foods [5]. 

Fatty acids influence health, well-being, and risk of disease. Traditionally the health impact of fatty acids was thought to be limited to cardiovascular disease. However, it is now clear that fatty acids influence a wider range of other diseases, including metabolic diseases such as type 2 diabetes, inflammatory diseases, and cancer [6].

Lipids are vital components of cells. They act as structural components of cell membranes and as storage products. All the lipids in higher organisms, including plants, contain significant quantities of polyunsaturated fatty acids (PUFA). The two principal families of PUFA that occur in nature, the n6 and the n3 fatty acids, are biosynthetically derived from linoleic acid (18:2 n6, LA) and α-linolenic acid (18:3 n3, ALA), respectively. Both fatty acids are synthesized in plants, but not in animal tissues; therefore they are essential dietary components (known as essential fatty acids, EFAs) and must be included in the human diet, in part by consuming plants [7,8,9]. In addition, n3 and n6 are required for growth, reproduction, good health, and to prevent cardiovascular diseases [10]. The ratio of these fatty acid families is important to our health. A ratio between 1 and 4:1 for n6 to n3 PUFA is now recommended by the WHO; however, the typical Western diet has a ratio between 15.0 and 16.7:1 [11]. This results shows a deficiency in n3 fatty acids [11]. Therefore, identifying sources of PUFAs within plants is of great importance [12]. Borage has been reported to contain these essential fatty acids [13].

Borage (*Borago officinalis* L.) is an herbaceous annual plant widely distributed beyond its original habitat in the Mediterranean region [14]. Traditionally, borage has been cultivated for culinary and medicinal uses; currently it is produced commercially as an oilseed crop. This is because borage is one of the best-known sources of γ-linolenic acid (18:3 n6, GLA), an unusual fatty acid in plants, highly appreciated because of its nutritional, cosmetic and medical benefits [15]. Besides, borage is also consumed as a vegetable. Consumption of borage as a vegetable has been reported in several countries: in the German region of Hessen (as an ingredient in green sauce), in the Italian region of Liguria (as an ingredient of the famous preboggion), in Crete, France, and Great Britain [16]. Borage is also extremely popular in Spanish cuisine, specifically in the regions of Aragon, La Rioja, and Navarra (Ebro Valley) [17]. According to statistics from the Spanish Ministry, in 2019, 161 hectares of land in Spain produced 6.603 tons of borage [18]. White flowers and the absence of anthocyanin pigments in green parts characterize the plant material cultivated in those regions. 

The parts of the plant usually consumed in Spain are the basal leaf petioles. They have to be harvested after the plant is fully developed, but before flowering occurs. In this way, the borage harvest period begins when the plants are at the end of their vegetative growth and ends at the floral induction stage. To facilitate their consumption, the borage industry commercializes only the petioles of the plants, removing the leaf blades and generating, as a result, a great amount of by-product. In the early stages of plant growth, 60% of the plant is composed of leaf blades and the remaining 40% of petioles [13], although these percentages can fluctuate depending on the type of borage and on the growing conditions. Nowadays, the by-product of the borage agri-food industry is used as animal feed, in particular to feed fighting bulls [17]. Alternatively, this by-product could be used to economically produce healthy products once their nutraceutical composition has been evaluated. Borage leaves contain pyrrolizidine alkaloids (PAs); this has raised concern about its uses as a vegetable [15]. Although the safety of borage has been questioned due to the presence of PAs, no case reports on *Borago officinalis* suggest that ingestion of borage causes harm. Nevertheless, Lozano-Baena et al. [4] verify the health benefits of Spanish traditionally consumed *Borago officinalis* plants and a review and quality assessment of case reports of adverse effects has been recently conducted by Avila et al. [19], concluding that the consumption of borage is safe for health. 

Although the distribution of fatty acids has been previously assessed in borage [13,20,21,22,23], up until now, their variability and evolution in a collection of different genotypes considering the different organs of the plant commonly used as food had not been determined. 

Bioactive compounds of fruits and vegetables are greatly influenced by their genetic background [24,25,26,27], developmental stage, rate of growth, and environmental factors [28,29,30]. We present the first report providing the fatty acid contents of a borage collection (wild, cultivated, including both commercial varieties and landraces, and breeding lines), grown simultaneously under winter greenhouse conditions. 

The present study aims to assess the effects of genotype, harvesting time, and part of the leaf on the borage fatty acid composition to identify their potential as a source of bioactive compounds. Our hypothesis states the use of borage leaves (petioles and leaf blades) as nutraceuticals for human consumption, feeding animals, medicine and pharmaceuticals.

## 2. Materials and Methods

### 2.1. Plant Material Description and Experimental Design

Twelve borage (*B. officinalis*) genotypes originating from different sources were studied (Table 1): three Spanish commercial varieties marketed for vegetable production; one Canadian variety marketed for seed production; two Spanish selected lines from two different borage vegetable breeding programs; three landraces cultivated as a vegetable in Spain and three wild accessions from Australia, France, and Malta. Except for the commercial varieties and the breeding line from La Rioja, seeds were provided by the Spanish Vegetable Genebank of the Agrifood Research and Technology Centre of Aragón (BGHZ-CITA). Information about the genebank accessions regarding latitude, longitude and elevation of collecting site, biological status, or acquisition source and date, among others, and the availability of seeds can be found on the website https://bghz.cita-aragon.es/app/ (accessed on 2 December 2021), by using the National Inventory Code (see Table 1). The breeding line Rioja is also available in the genebank with some restrictions.

These genotypes were cultivated under greenhouse conditions, with termostatically controlled heater to avoid temperatures less than 8 °C, at the Technological College of Huesca (University of Zaragoza) facility, Huesca, Spain (42°07′03″ N, 0°26′47″ W and 488 m above sea level) using a randomized complete block design with three replications. 

Each replication consisted of four plants that were transplanted at the first true leaf stage on the 15th of November with a distance of 40 cm between plants and a row-to-row distance of 40 cm, equivalent to a crop density of 6.25 plants m^−2^ at green-house conditions. Soil preparation, planting, and other agronomic practices were carried out uniformly following the standard growth borage practices. The soil was a clay loam, calcareous (30%), slightly saline (5.1 dS/m), and alkaline (pH 8), with a relative low amount of organic matter (1.5%). The water supply was based on a demand drip irrigation system. 

Forty-eight leaves per genotype were harvested (four leaves for each of the four plants and replication) at two development stages. The first stage (120 days after sowing, das) corresponds to the commercial harvesting time or optimal growth stage to consume borage as a vegetable for white flowering varieties (stage 2.0 to 2.4 of the Simpson’s code of development [31]) (Table 1). The second stage (150 das) corresponds to the start of the plants flowering process, broad flowering stage or seed maturation and abcision depending on the genotype (stage 3.0 to 5.n Simpson´s code of development [31]) (Table 1). The leaves from each genotype were divided into leaf blades and petioles and were collected to determine their fatty acid composition. Samples were dehydrated in a forced air oven at 60 °C for about 24 h, until constant weight. They were then ground and stored in three sealed dark envelopes according to their genotype. The envelopes were placed in a desiccator and kept at 4 °C.

### 2.2. Chemicals Reagents

Fatty Acid Methyl Esters (FAMEs) (OL—oleic acid, LA—linoleic acid, GLA—γ-linolenic acid, ALA—α-linolenic acid) were purchased from Sigma Chemical Company (St. Louis, MO, USA). Only analytical grade chemical reagents were used (Merck, Darmstadt, Gesse, Germany, and Panreac, Castellar del Vallès, Barcelona, Spain).

### 2.3. Determination of Fatty Acids Composition

To determine fatty acid composition, direct transesterification of the sample to fatty acid methyl esters (FAMEs) under alkaline conditions, followed by extraction and analysis by gas chromatography was carried out. The method was adapted from ISO 12966-2 [32].

Approximately 100 mg powdered material from each sample was transferred to a test tube and 3 mL of a sodium methoxide solution (5% of metal sodium in methanol-isopropanol (80:20)) was added. The mixture was shaken for 30 s and left to react for 20 min at room temperature. Three consecutive extractions were performed with 3 mL of hexane to extract the FAMEs. The total extract was stored in amber vials and kept in a refrigerator until the chromatographic analysis was conducted. Prior to injecting the samples into the chromatograph, the extracts were filtered.

The fatty acid composition was analyzed by a Hewlett Packard Gas Chromatograph (model 4890) equipped with a flame ionization detector (FID) and a capillary column, HP Innowax (60 m length × 0.32 mm i.d. × 0.25 μm film thickness). The temperatures of the injector and detector were 250 °C and 300 °C, respectively. The initial column temperature was 100 °C, and was raised to 255 °C at a rate of 10 °C/min, the final temperature was held for 20 min. Hydrogen was used as a carrier gas at a flow rate of 1 mL/min. The samples were injected manually with a split ratio of 1:30. Analysis was performed in duplicate.

The identification of chromatographic peaks was performed by comparing the retention times with the standards OL, LA, GLA, and ALA. Other peaks different from these standards were identified by comparing the elution order with those reported in the literature consulted [13,21,23,24,29,33,34,35,36].

The fatty acid profile was expressed as the percentage of individual fatty acids area of relative to total area of all identified fatty acids in the sample. Besides, GLA and ALA contents were estimated in mg g^−1^ of dry weight. Calibration curves were plotted (peak area versus concentration) using diluted solutions in hexane for each fatty acid methyl ester with a concentration range between 4.0 and 200.0 µg mL^−1^.

### 2.4. Statistical Analysis

The results were analyzed from the mean of determinations for duplicate samples prepared for each genotype. Data was expressed as the mean ± the standard deviation (SD). Means were compared using the one-way analysis of variance (ANOVA) followed by a post hoc Tukey-b test to construct homogeneous groups. The differences between individual means were deemed to be significant at *p* < 0.05. Multivariate analysis of the principal component analysis (PCA) was performed to extract new features (principal components) which were not redundant combinations and account for most of the data variability. All analyses were performed using the SPSS statistical package (SPSS for Windows, version 16.0).

## 3. Results and Discussion

### 3.1. Fatty Acids Composition

**Figure 1:**
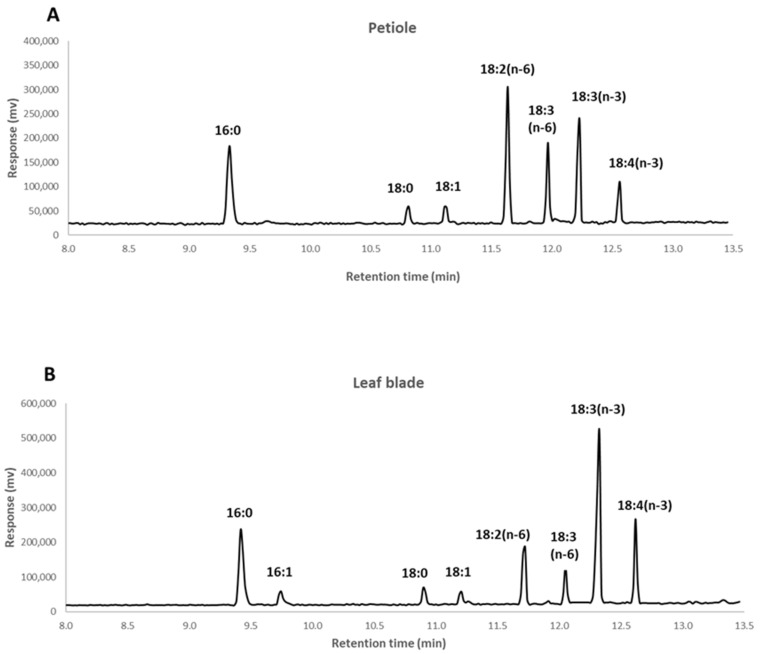
Representative chromatogram of fatty acid methyl esters from the lipid extract of petiole ((**A**), corresponding to sample: BL-RI_120das) and leaf blade ((**B**), corresponding to sample: BL-RI_150das) of borage, using gas chromatography.

The study identifies eight fatty acids for both the leaf blades and the petioles: palmitic acid (PA, 16:0), palmitoleic acid (PLA, 16:1), stearic acid (STA, 18:0), oleic acid (OA, 18:1), linoleic acid (LA,18:2, n-6), γ-linolenic acid (GLA, 18:3, n-6), α-linolenic acid (ALA, 18:3, n-3) and stearidonic acid (SDA, 18:4, n-3) (Figure 1).

For each of the eight fatty acids identified, there were significant differences (*p* < 0.05) for all 12 borage genotypes. The fatty acid composition depended on the plant’s developmental stage at the sampling date (120 or 150 days after sowing -das) and the part of the leaf that was being analyzed (leaf blade or petiole). Except for the PLA and STA fatty acids, which did not present differences between sampling dates (*p* = 474 and *p* = 0.898, respectively), the interaction among genotypes, sample, and date of sampling were also statistically significant (*p* < 0.05).

In general, the results showed that the fatty acid profile in the different parts of the leaves is characterized by the prevalence of five fatty acids (Table 2, Table 3, Table 4 and Table 5): PA, LA, ALA, GLA and SDA. These fatty acids account, on average, for approximately 90% of the total fatty acids, in both leaf blades and petioles. The amount of each of PLA, STA, and OA could be considered testimonial, with percentages below 5%. Other minority peaks, with an area less than 0.25%, were observed, but as all of them represent a percentage less than 1.5% of the total area, they were not considered in this study. 

PA was the most common fatty acid found in the leaf blades and petioles. This agrees with previous studies, which reported PA as one of the most common saturated fatty acids found in the green tissues of plants [37]. In our study, all borage increased the amount of PA in their leaf blades between harvest dates (18.9% to 25.6% on average). This was probably due to the old age of the tissues and was slightly constant in petioles (24.3% to 24.9%). Different behavior between the leaf blades and the petioles could be due to anatomical and physiological differences. Leaf blades are mainly photosynthetic tissues so their degradation implies the increase of saturated fatty acids. Petioles are transport tissues and lack foliar lamina; aging does not affect their lipid evolution equally. This aspect is more evident in white flowered borage, which were selected for their long petiole length, therefore minimizing the leaf blade. 

LA and ALA are members of the essential fatty acid groups, so called because they are essential dietary requirements for all mammals. The amount of LA is higher in petioles than in leaf blades and decreases between sampling dates (25.1% to 18.6% in petioles vs. 11.2% to 10.6% in leaf blades). ALA is the most common fatty acid in leaf blades, representing 34.7% and 37.6% at the first and second sampling dates, respectively. These ALA leaf blades data are higher than data shown in petioles (19.4% at 120 das to 27.6% at 150 das).

The SDA fatty acid, a metabolite of ALA via D6-desaturase, was detected in lower percentages (14.6% to 12.1% in leaf blades and 7.3% to 9.1% in petioles) than that obtained by Peiretti et al. [22] or by Stahlër et al. [33], 24% and 25% respectively. Additionally, GLA, a metabolite of LA, also via D6-desaturase, is present in both parts of the leaves in percentages similar to those of SDA. Its quantity decreases between sampling in leaf blades (11.0% to 4.6%) and petioles (14.7% to 9.9%). Only a few species such as evening primrose, black currant, and some *Boraginaceae* family members contain products of the D6-desaturase enzyme. As Stahlër et al. [33] pointed out, in these species D6-desaturases enzymes coexist in different organelles, chloroplast, and endoplasmic reticulum, with different specificities. Both metabolic pathways of LA and ALA (n-6 and n-3 respectively) do not compete for D6-desaturase and both GLA and SDA could be synthesised independently in different cell organelles [33]. This metabolic pathway justifies the presence of SDA and GLA found in borage leaves.

Extensive research has been conducted on the fatty acid composition of the borage’s green parts. Fatty acids shown in this study could be attributed to the heterogeneity of the plant material (wild or cultivated), the part of the plant being analysed, the sampling dates, the different growing cycle stages, the environmental factors (light, nutrition, and agronomic practices) [20,21,22,23,33,34,35,36,38,39], or the preparation of samples (f.e. oven-dried instead of freeze-dried). Only Del Río-Celestino et al. [13] studied the fatty acid content in leaf blades and petioles in white- and blue-flowered borage. Their results differ from ours both qualitatively and quantitatively. These authors detected a high proportion of myristic acid (14:0) in petioles (56% in blue flowered borage and 27% in white flowered). This fatty acid is not detected in our samples. Similarly, we did not detect erucic acid (22:1 n-9) but it was found in low concentrations (0.3–0.9%) by Del Río-Celestino et al. [13]. These differences could be explained by the following considerations: the genotype and, in particular, the environmental conditions. In fact, plants vary their membrane lipid composition to vary their cell fluidity and permeability. This allows them to adapt to changing environmental conditions [33,34]. In our case, all plants were grown under cool greenhouse conditions. Therefore, differences observed between borage can only be due to genotype.

### 3.2. Fatty Acids Groups

**Figure 2:**
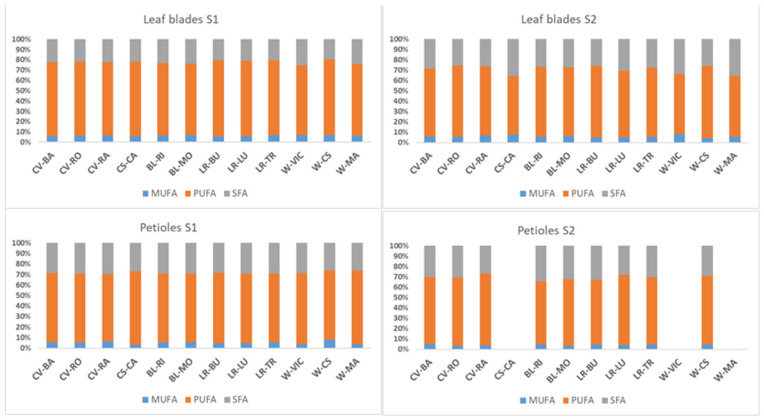
Polyunsaturated fatty acids (PUFA), Monounsaturated fatty acids (MUFA) and Saturated fatty acids (SFA) distribution, expressed as percentage, in borage limbs and petioles sampled at 120 days after sowing (S1) and 150 days (S2) of 12 borage accessions (see Table 1). Samples without values mean sessile leaves at this plant-growing period.

Results for the total saturated fatty acids (SFA), monounsaturated fatty acids (MUFA), and polyunsaturated fatty acids (PUFA) of the borage leaf blades and petioles sampled at 120 das and 150 days are shown in Figure 2. Unsaturated fatty acids (UFA) predominate over SFA. The fatty acid composition of the whole borage plant was characterised by a high percentage of PUFA in leaf blades (71.5% 120 das and 64.9% 150 das) and petioles (66.5% 120 das and 65.2% 150 das). This data agrees with Mhamdi et al. [23], who found similar percentages and types of fatty acids in the foliar tissues of borage. Similarly, Abbaszadeh et al. [28], also recorded similar data in *Echium* sp. leaves, a wild edible species of the *Boraginaceae* family. In all the borages and in their samples, PUFA predominates over MUFA due to the contribution of LA, ALA, GLA, and SDA in front of OA and PLA. In all borages, MUFA represented less than 10% of total fatty acids. SFA included the main contribution of palmitic acid and stearic acid (limbs 22.1% to 29.2% and petioles 28.4% to 30.5% for first to second sampling date, respectively). 

Figure 2 presents the relationship between PUFA and SFA. SFA increased as plants aged and PUFA decreased for both parts of the leaves as plants aged. This tendency is present in all borages. Interestingly, the relation between PUFA/SFA and n6/n3 for nutritional quality shows health benefits. The PUFA/SFA ratio (data not shown) varied from 2.14 in petioles of the second sampling date, to 3.23 in leaf blades of the first sampling date. According to Pereira et al. [35] this ratio should be higher than 0.45. In reference to n6/n3 (data not shown), the ratio varied from 0.31, in leaf blades of the second sampling date, to 1.49, in petioles of the first sampling date. According to Simopoulus [40] this ratio should be lower than 4.0. These results reinforce the fact that borage leaves have a good nutritional quality.

### 3.3. Borage Germplasm Diversity

To investigate the relationships between borage plant germplasms, we performed a multivariate data analysis based on the fatty acid composition. Principal component analysis allowed us to decrease the number of descriptors associated with the data matrix of analyzed variables (i.e., the fatty acids, the part of the leaf analyzed, and the growth stage). Palmitoleic acid was not considered because it was only detected in extremely low percentages in some petiole samples. Pearson correlation coefficients between fatty acid variables showed positive correlations between ALA and SDA (r = 0.826) and between LA and GLA (r = 0.749). On the other hand, a negative correlation between the first group and the second group; that is ALA-LA (r = −0.958), ALA-GLA (r = −0.810), SDA-LA (r = −0.867), and SDA-GLA (r = −421) was observed. This means that borages with high levels of ALA and SDA have fewer amounts of LA and GLA. Wannes et al. [41], who analyzed the lipid and fatty acid distribution in leaves and seeds during borage development, found similar correlations. In plants, ALA and LA fatty acids are related to two metabolic pathways, n-3 and n-6 respectively. LA is a substrate for the biosynthesis of ALA and GLA. As Griffiths et al. [21] pointed out, both D15 and D6-desaturase compete for LA. If D15-desaturase activity predominates, ALA (and subsequently SDA) will be synthesized. Then, n-3 fatty acids predominate. Contrarily, if D6-desaturase activity predominates, n-6 pathways yield and consequently GLA is produced. In general, ALA is the predominant fatty acid in the green tissues of plants because they lack D6-desaturase. In the case of borage, the presence of this unusual enzyme in plants allows the synthesis of both GLA and SDA.

Principal component analysis has resulted in a two principal component model explaining 84.4% of the total variance. The first principal component (PC1) had the highest eigenvalue, 3.65, and accounts for 60.2% of the total data variability, the second one (PC2) had an eigenvalue of 2.94 and accounted for 24.2% of the variance. 

**Figure 3:**
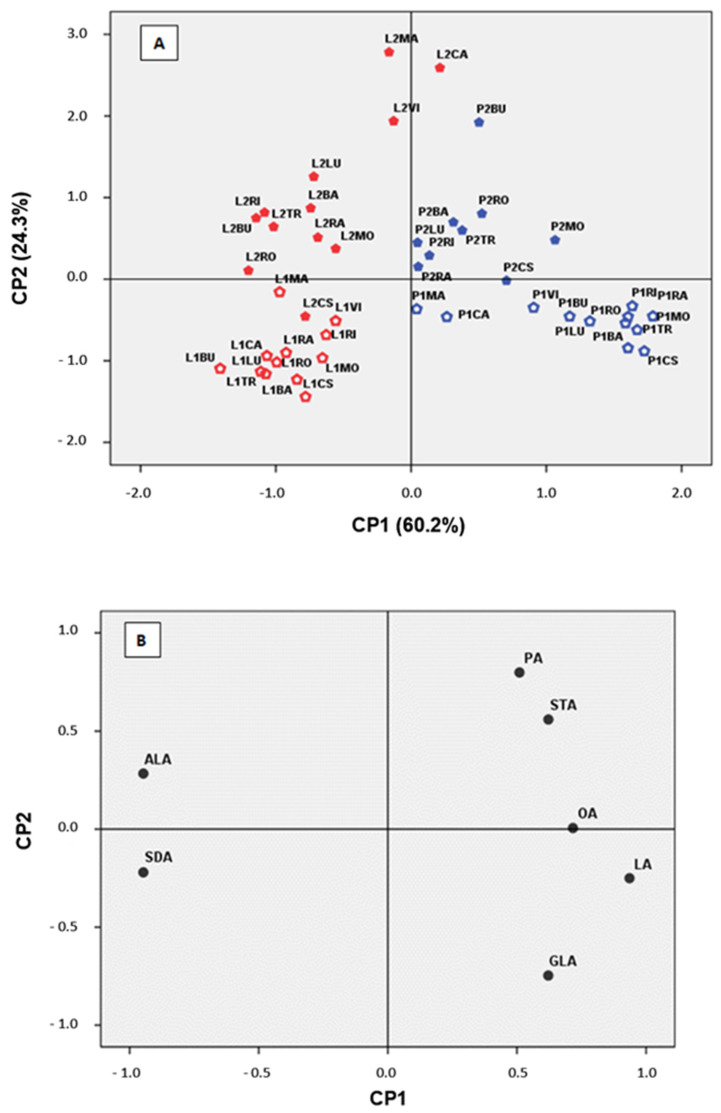
Similarities among 12 borage accessions (BA, RO, RA, CA, RI, MO, BU, LU, TR, VI, CS, and MA) evaluated based on fatty acids at two harvest dates: 120 (1) and 150 (2) days after sampling and two parts of the leaf: leaf blades (L) and petioles (P) represented in (**A**) the two first components (first component, x-axis; second component, y-axis) of the principal components analysis (60.2% and 24.3% of the total variation, respectively) and (**B**) the similarities among the seven fatty acids (ALA, SDA, PA, STA, GA, LA, and GLA).

Specific patterns of correlation between the variables being tested can be visualised by comparing loading plots of the PCs. Figure 3A,B show the score plot and the loading plot of PC1-PC2 for the borage samples that were analysed. In this analysis, all the fatty acids influence the model. Component 1 is substantially influenced by ALA and SDA (loading negative high), LA (loading positive high), and OL (less substantially). Component 2 is influenced mainly by PA (positive) and GLA (negative).

Figure 3A shows four distinct groups, where differences in fatty acid composition between the sampling dates and the parts of the leaf are evident. These results agree with Sewon and Tyystjarvi [20], who pointed out that the variation in fatty acid composition is related to physiological factors associated with the plant’s developmental stage. Between sampling dates, PA and STA increased while mainly decreasing GLA. It is likely that plants from the second harvest, which are in a late developmental stage, transformed GLA in their sugar to accumulate in seed, as Salisbury et al. [42] pointed out.

Leaf blades (the by-product of the borage industry) and petioles (the most common edible organ of the plant) are separated due to their content of ALA and SDA in front of LA, GLA and OA. It was established that chloroplasts in green photosynthetic tissue have high levels of D15-desaturase, the enzyme responsible for desaturation of the LA to ALA [33]. In this sense, the physiology of the leaf blade and the petioles could be the cause for differences in the amounts of fatty acids in the different parts of the leaf.

Figure 3 also provides evidence for the differences between white- and blue-flowered borage. Petioles sampled at 120 das from white-flowered borage located nearby, were influenced by LA, GLA and OA. In contrast, blue-flowered ones (P1VI, P1CA and P1MA) were separated because of ALA and SDA contributions. For petioles of 150 das, no data exist for blue-flowered borage because the leaves were sessile at this developmental stage. PCA also provided evidence of some morphological singularities observed during cultivation of LR-BU, BL-MO and W-CS. Spanish landrace LR-BU is characterised by its large size and other specific developmental traits that differentiate it from other white-flowered borage. The selected line of BL-MO was obtained after a breeding program. This allowed for the flowering stage to be delayed and for longer petioles to be obtained. These petioles differ from those of other white-flowered borage. The French wild accession, W-CS, presented a spontaneous flowering delay when compared to the remaining Spanish plants. 

Leaf blades at 120 das differed from those at 150 das due to the contribution of SFA in PC2. The limbs of second samples confirmed the genetic fatty acid behavior observed in the petioles of white- and blue-flowered accessions. When leaf blades and petioles were analyzed separately, these results were reinforced. Sales et al. [43], who studied some of the genotypes used in this work, also described a similar trend in borage germplasm after using molecular tools.

### 3.4. Relevance of Borage Leaves Fatty Acids in Diet

The major polyunsaturated fatty acids identified in borage leaves (LA, ALA, GLA, and SDA) are especially relevant from a nutritional and/or nutraceutical point of view because of their biological value. Mammals cannot synthesize LA and ALA, and GLA and SDA are essential to the daily diets of people with certain disease disorders [44]. Diseases such as cardiovascular pathologies, inflammatory processes, viral infections, autoimmune diseases and some types of cancer cause D6-desaturase inhibition and GLA and SDA become a rate-limiting step in the production of long-chain (C > 20) fatty acids, especially those in the n-3 family, which are vital for human health. The presence of LA, ALA, GLA and SDA in borage leaves could be a good potential source of these fatty acids. Consumption of borage would therefore help to prevent these pathologies.

To establish the relevance of borage leaves in our diet, the content of ALA and GLA were determined. Both were quantified in the petioles, traditionally consumed in the Spanish cuisine of the Ebro Valley, and leaf blades, the by-product of the food industry (that could be used in animal diets). In fact, due to natural and economic reasons, borage leaf blades may become an ingredient in animal feed and they could also become an alternative to pharmacological treatments administered to ruminants and horses undergoing stress, as suggested by Peiretti et al. [22]. The analysis was also carried out at two sampling dates (120 das and 150 das), to identify the best time to harvest borage to achieve maximum benefits from it.

**Figure 4:**
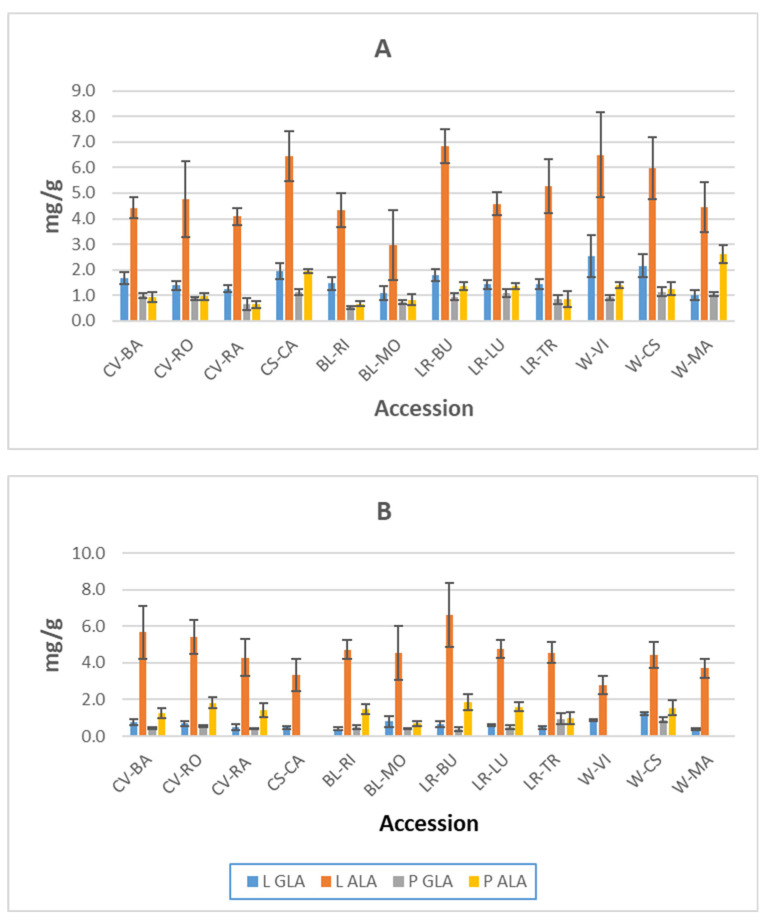
γ-linolenic acid (18:3 n6, GLA) and α-linolenic acid (18:3 n3, ALA) contents (mg/g of dry weight) in leaf blades (L) and petioles (P), sampled 120 days after sampling (**A**) and 150 days after sampling (**B**) (LOQ = 0.260 mg/g and LOD = 0.078 mg/g). Samples without values mean sessile leaves at this plant-growing period. Samples without values mean sessile leaves at this plant-growing period.

Figure 4 highlights the amount of ALA found in all genotypes and shows it to be higher in leaf blades than in petioles. For leaf blades, ALA ranged from 6.84 mg/g dw (LR-BU, 120 das) to 2.79 mg/g dw (W-VI, 150 das) while in petioles it ranged from 2.61 mg/g dw (W-MA, 120 das) to 0.65 mg/g dw (BL-MO, 150 das). Del Rio-Celestino et al. [13] reported a similar trend. They found this fatty acid predominates, and observed that it was higher in leaf blades than in petioles. Nevertheless, to our knowledge, this is the first time that GLA has been quantified in borage leaves, and only Baya et al. [38] and Borowi et al. [39] have quantified ALA in borage stalk leaves. They obtained values of 11.55 mg/g in dry weight and 0.21 mg/g in fresh weight, respectively. Compared to other vegetables, the borage ALA content is higher than previously reported for cultivated spinach (0.14 mg/g) and lettuce (0.10–0.12 mg/g) but comparable to that of the edible wild plant purslane (4.05 mg/g) [34]. According to Simopoulos [40], modern agriculture with its emphasis on production has decreased the n3 fatty acid content in many foods. In fact, the lowest value obtained for ALA corresponds to BL-MO, a breeding line selected for quality and high productivity [45]. 

Some studies have shown evidence of ALA being associated with cardiovascular disease, type 2 diabetes, and fracture risk [46]. Although an important source of ALA is the consumption of fatty fish, it may not be practical for those who are concerned with the unsustainability of marine food-sources or who avoid eating fish for a variety of reasons. In that way, ALA-rich plant sources, such as borage, are more abundant and may serve as a suitable alternative. 

Figure 4 shows that in general the amount of GLA is higher in leaf blades than in petioles. For leaf blades GLA ranged from 2.54 mg/g dw (W-VI, 120 das) to 0.38 mg/g dw (W-MA 150 das) whereas in petioles it ranged from 1.14 mg/g dw (W-CS 120 das) to 0.38 mg/g dw (LR-BU, 150 das). Sewon and Tyystjarvi [20] found a variation between 2.6 mg/g dw and 0.5 mg/g during the wild borage growing period, this trend is also consistent with our results, since we found higher GLA content at the first sampling date. Seeds of *Boraginaceae* species constitute a good source of GLA-rich oils, suitable to be marketed by the pharmaceutical and food industries [47,48]. Our results illustrate that borage leaves also contain GLA, although in much smaller quantities. 

The effects of harvest time (120 das and 150 das) on fatty acid composition allow us to know the best time to harvest borage to achieve its maximum health benefits. It was concluded that the content of both ALA and GLA decreased due to aging. Considering the health benefits, it is better to harvest at 120 das. 

During the last century, the intake of n3 fatty acids has decreased while that of n6 fatty acids and saturated fats has increased [49]. Our results showed that borage leaves are characterised by an adequate PUFA/SFA proportion and a low n6:n3 ratio, which support its significant dietary nutraceutical value.

Data on fatty acid composition can be used for the calculation of lipid quality indexes, including the atherogenetic index (AI) and the thrombogenic index (TI) [50,51]. These indexes take into account the different effects that single fatty acids might have on human health and in particular on the probability of increasing the incidence of pathogenic phenomena, such as atheroma and/or thrombus formation. The lipid quality index determined in this study, corresponding to AI, showed mean values for leaf blades of 0.28 (120 das) and 0.42 (150 das), and for petioles 0.40 (120 das) and 0.43 (150 das). Moreover, the TI index showed mean values for leaf blades of 0.14 (120 das) and 0.18 (150 das) and for petioles 0.28 (120 das) and 0.23 (150 das). Those AI and TI values for borage leaf blades and petioles can be considered as low, which is in agreement with literature reports [50,51]. Accordingly, the nutraceutical borage value is reinforced based on these data.

## 4. Conclusions

This study shows the effects of genotype, time of harvest, and part of the leaf on the borage fatty acid composition. The results have shown a great genetic variability in borage based on the leaves’ fatty acid composition. This aspect is relevant because it is possible to design borage-breeding programs to increase certain essential fatty acids for a healthy diet.

The main polyunsaturated fatty acids identified LA, ALA, GLA, and SDA, which account for approximately 70% of fatty acids, support the nutraceutical interest of borage leaves for animal and human consumption because they have the potential to prevent cardiovascular diseases, cancer, and infectious diseases. Specifically, we can highlight the presence of GLA and SDA, which are fatty acids that are not commonly found in plants, and LA and ALA, which are considered essential fatty acids for mammals and must be a part of their diet. The relationship between saturated and polyunsaturated fatty acids and the ratio of the polyunsaturated families n6 and n3 reinforce the borage nutritional value. Based on the clustering, white and blue-flowered borage were differentiated clearly. For example, blue-flowered genotypes W-VI, CS-CA, and W-MA stood out by their high content of ALA and SDA. These richer sources of PUFA can’t be adapted to large-scale production as a vegetable due to their morphological and physiological traits. Out of these three accessions, two are wild genotypes and the other one is grown for the production of seed oil. Nevertheless, they can be exploited in borage breeding programs for edible purposes. For this reason, it is interesting to encourage the preservation of these accessions as a source of variability of nutraceutical compounds.

The findings of this study allow us to make some nutritional considerations. Petioles from young plants should be harvested for diets with higher n6 fatty acids, while older plants produce petioles with a great amount of n3 fatty acids. The higher content of ALA in the leaf blades, the part of the leaf that is generally discarded as a by-product by the borage industry, gives them a great dietary potential as they have significant health benefits. Therefore, the use of borage leaf blades for human consumption, animal feeding, medicine, and pharmaceuticals should be promoted.

The results of this study regarding the borage´s fatty acid, allow us to confirm the potential of borage leaves, both the petioles and the leaf blades, as a source of nutraceutical compounds.

## Figures and Tables

**Table 1:** Borage (*Borago officinalis* L.) genotypes evaluated and plant stages during sampling: 120 and 150 days after sowing (d.a.s.).

Acronym	Accession	Source	Origin	Spanish National Inventory Code	Flower Color	Stage 120 d.a.s. *	Stage 150 d.a.s. *
CV-BA	Batlle	Commercial (vegetable)	Spain	-	White	2.2	3.1
CV-RO	Rocalba	Commercial (vegetable)	Spain	-	White	2.2	5.0
CV-RA	Ramiro Arnedo	Commercial (vegetable)	Spain	-	White	2.2	3.1
CS-CA	Richters herbs	Commercial (seed)	Canada	-	Blue	4.n	5.n
BL-RI	Rioja	Breeding line (Research center)	Spain	-	White	2.2	4.n
BL-MO	Movera	Breeding line (genebank)	Spain	NC104614	White	2.1	3.1
LR-BU	Buñuel	Landrace (genebank)	Spain	NC078140	White	2.4	5.n
LR-LU	Luceni	Landrace (genebank)	Spain	NC019518	White	2.1	5.n
LR-TR	Triste	Landrace (genebank)	Spain	NC073372	White	2.1	5.n
W-VI	Victoria	Wild (genebank)	Australia	NC078139	Blue	4.n	5.n
W-CS	Chateau succinio	Wild (genebank)	France	NC078142	White	2.0	4.n
W-MA	Malta	Wild (genebank)	Malta	NC096632	Blue	4.n	5.n

* Simpson’s code of development. - The accession is not listed in the National Inventory.

**Table 2:** Fatty acid composition, expressed in percentage of total fatty acids, of petioles of 12 borage accessions sampled at 120 days after sowing (Mean ± SD (n = 6)).

Acc.	Palmitic C16:0	Palmitoleic C16:1	Stearic C18:0	Oleic C18:1	Linoleic C18:2	GLA C18:3(n-6)	ALA C18:3(n-3)	Stearidonic C18:04
CV-BA	24.85 ± 1.19 a	nd	3.85 ± 0.47 b	5.35 ± 0.69 bc	27.62 ± 3.10 abc	16.45 ± 1.54 a	15.49 ± 1.58 fg	6.41 ± 0.70 cd
CV-RO	25.06 ± 0.59 a	nd	4.09 ± 0.53 ab	5.41 ± 1.08 bc	27.78 ± 1.25 abc	14.90 ± 0.95 bc	16.14 ± 0.95 efg	6.62 ± 0.58 cd
CV-RA	25.64 ± 1.63 a	nd	4.13 ± 1.37 ab	6.16 ± 0.67 b	27.58 ± 5.13 abc	15.10 ± 2.08 abc	15.26 ± 5.50 fg	6.13 ± 2.67 de
CS-CA	23.00 ± 1.02 bc	*	4.00 ± 0.86 ab	3.27 ± 0.93 e	18.72 ± 1.04 g	13.73 ± 0.65 c	27.09 ± 1.16 b	10.19 ± 0.77 a
BL-RI	25.03 ± 2.64 a	*	4.15 ± 1.00 ab	5.44 ± 1.66 bc	28.73 ± 2.44 ab	13.69 ± 0.83 c	17.54 ± 4.56 de	5.42 ± 1.48 e
BL-MO	25.06 ± 1.02 a	nd	4.29 ± 0.74 ab	5.46 ± 0.88 bc	26.41 ± 1.86 cd	15.51 ± 0.98 ab	16.84 ± 2.32 def	6.42 ± 0.51 cd
LR-BU	24.04 ± 1.69 ab	*	4.18 ± 0.80 ab	4.61 ± 0.77 cd	25.00 ± 1.14 de	14.41 ± 0.35 bc	20.61 ± 1.90 c	7.15 ± 1.02 bc
LR-LU	25.56 ± 2.76 a	nd	3.72 ± 1.02 b	4.67 ± 0.46 cd	26.98 ± 1.57 bc	14.37 ± 1.03 bc	18.22 ± 3.35 d	6.48 ± 1.39 cd
LR-TR	25.23 ± 2.56 a	nd	4.03 ± 0.51 ab	5.32 ± 0.41 bc	28.98 ± 0.98 a	15.23 ± 0.69 ab	14.85 ± 2.01 g	6.37 ± 0.56 cd
W-VI	24.75 ± 1.99 a	*	4.08 ± 0.75 ab	3.93 ± 0.70 de	22.86 ± 1.08 f	14.35 ± 0.92 bc	22.16 ± 1.61 c	7.87 ± 1.71 b
W-CS	21.51 ± 1.23 c	*	4.58 ± 0.54 a	7.91 ± 1.56 a	23.72 ± 0.71 ef	16.45 ± 0.47 a	18.08 ± 2.10 d	7.74 ± 0.89 b
W-MA	22.31 ± 3.09 c	*	3.91 ± 0.83 b	3.59 ± 0.91 e	17.17 ± 1.87 g	12.24 ± 1.67 d	30.00 ± 3.17 a	10.78 ± 2.50 a
Mean	24.34	-	4.08	5.09	25.1	14.70	19.36	7.30
Range	(21.51–25.64)	-	(3.72–4.58)	(3.27–6.16)	(17.17–28.98)	(12.24–16.45)	(14.85–30.00)	(5.42–10.78)

nd: non detected; * non considered (average less than 0.25%); - non calculated. Values within each column followed by different letters are significantly different at *p* < 0.05.

**Table 3:** Fatty acid composition, expressed in percentage of total fatty acids, of petioles of 12 borage accessions sampled at 150 days after sowing (Mean ± SD (n = 6)).

Acc.	Palmitic C16:0	Palmitoleic C16:1	Stearic C18:0	Oleic C18:1	Linoleic C18:2	GLA C18:3(n-6)	ALA C18:3(n-3)	Stearidonic C18:04
CV-BA	25.49 ± 1.39 ab	1.81 ± 0.41	4.69 ± 0.70 b	3.35 ± 0.60 ab	17.74 ± 1.46 bc	9.37 ± 0.90 b	27.49 ± 2.15 ab	10.06 ± 1.63 a
CV-RO	23.84 ± 1.96 bc	nd	4.98 ± 0.20	3.36 ± 0.67 ab	19.74 ± 2.83 b	8.84 ± 1.17 b	30.32 ± 2.97 ab	8.92 ± 2.06 abc
CV-RA	22.66 ± 0.63 c	nd	4.31 ± 0.27 b	3.78 ± 0.51 ab	17.74 ± 0.50 bc	9.60 ± 0.59 b	31.06 ± 1.25 a	10.86 ± 0.88 a
CS-CA	sl							
BL-RI	23.49 ± 0.99 bc	1.17 ± 0.26	4.13 ± 0.26 b	3.38 ± 0.34 ab	16.05 ± 0.41 c	8.74 ± 1.38 b	26.65 ± 1.53 bc	9.23 ± 1.80 abc
BL-MO	27.56 ± 1.72 a	nd	4.77 ± 0.23 b	3.21 ± 0.51 ab	23.20 ± 1.06 a	12.97 ± 0.72 a	20.83 ± 1.44 d	7.46 ± 0.95 c
LR-BU	27.54 ± 1.83 a	1.52 ± 0.26	6.05 ± 0.20 a	3.39 ± 0.50 ab	17.56 ± 1.48 bc	5.90 ± 0.68 c	30.21 ± 1.53 ab	7.82 ± 0.90 bc
LR-LU	23.49 ± 0.81 bc	1.09 ± 0.24	4.56 ± 0.27 b	2.74 ± 0.32 b	17.74 ± 0.30 bc	9.44 ± 0.58 b	31.28 ± 1.11 a	9.66 ± 0.98 ab
LR-TR	25.40 ± 1.31 ab	1.55 ± 0.23	4.50 ± 0.29 b	3.23 ± 0.32 ab	18.20 ± 0.62 bc	9.71 ± 0.98 b	27.36 ± 1.80 ab	10.06 ± 1.58 a
W-VI	sl							
W-CS	24.33 ± 1.39 bc	0.61 ± 0.20	4.88 ± 0.36 b	4.22 ± 0.36 a	18.50 ± 1.03 bc	14.01 ± 0.77 a	23.42 ± 1.49 cd	10.04 ± 1.32 a
W-MA	sl							
Mean	24.87	1.25	4.73	3.40	18.57	9.88	27.63	9.14
Range	(22.66–26.89)	(nd−1.81)	(4.13–5.78)	(2.74–417)	(16.05–23.20)	(8.74–13.95)	(20.83–31.57)	(7.44–10.86)

nd: non detected; sl: sessile leaf; Values within each column followed by different letters are significantly different at *p* < 0.05.

**Table 4:** Fatty acid composition, expressed in percentage of total fatty acids, of leaf blades of 12 borage accessions sampled at 120 days after sowing (Mean ± SD (n = 6)).

Acc.	Palmitic C16:0	Palmitoleic C16:1	Stearic C18:0	Oleic C18:1	Linoleic C18:2	GLA C18:3(n-6)	ALA C18:3(n-3)	Stearidonic C18:04
CV-BA	19.11 ± 1.64 bcd	2.85 ± 0.51 cd	2.97 ± 0.28 d	3.21 ± 0.49 b	11.69 ± 1.00 ab	12.55 ± 1.57 a	33.15 ± 2.79 cde	14.48 ± 0.56 abc
CV-RO	18.81 ± 0.45 bcde	3.08 ± 0.42 bc	2.99 ± 0.16 d	3.36 ± 0.48 b	11.26 ± 0.60 abc	10.58 ± 1.48 bc	35.03 ± 1.72 bcd	14.88 ± 1.25 ab
CV-RA	19.06 ± 0.84 bcd	3.07 ± 0.13 bc	3.17 ± 0.14 cd	3.24 ± 0.45 b	11.44 ± 0.18 abc	10.66 ± 1.33 bc	34.88 ± 1.09 bcd	14.47 ± 0.53 abc
CS-CA	18.84 ± 2.18 bcde	3.05 ± 0.23 bc	3.13 ± 0.56 cd	2.98 ± 0.51 b	9.89 ± 0.71 c	11.12 ± 3.22 abc	36.41 ± 8.63 ab	14.59 ± 2.79 abc
BL-RI	19.69 ± 1.61 bc	3.46 ± 0.09 ab	3.56 ± 0.08 abc	3.08 ± 0.12 b	11.89 ± 0.30 ab	11.68 ± 0.98 ab	34.33 ± 1.89 bcd	12.32 ± 0.93 d
BL-MO	20.09 ± 1.41 ab	3.41 ± 0.28 ab	2.95 ± 0.22 a	3.65 ± 0.37 ab	12.28 ± 1.24 ab	11.98 ± 0.69 ab	32.43 ± 2.29 de	13.21 ± 0.80 cd
LR-BU	17.16 ± 1.44 ef	3.28 ± 0.06 bc	3.11 ± 0.14 cd	2.16 ± 0.48 c	10.54 ± 0.42 bc	9.94 ± 0.57 c	37.88 ± 1.66 a	15.94 ± 0.63 a
LR-LU	17.91 ± 0.72 cdef	2.99 ± 0.08 bcd	3.06 ± 0.06 d	2.99 ± 0.05 b	11.33 ± 0.09 abc	11.08 ± 0.92 abc	35.62 ± 0.85 abc	15.03 ± 0.66 ab
LR-TR	17.59 ± 1.03 def	3.18 ± 0.19 bc	3.00 ± 0.24 d	3.63 ± 0.27 ab	11.38 ± 0.53 abc	9.75 ± 2.17 c	35.46 ± 1.60 abc	16.02 ± 0.99 a
W-VI	21.57 ± 0.93 a	3.74 ± 0.57 a	3.67 ± 0.48 ab	3.21 ± 0.20 b	10.87 ± 2.74 bc	12.00 ± 0.60 ab	31.13 ± 1.33 e	13.81 ± 0.57 bcd
W-CS	16.42 ± 1.33 f	2.54 ± 0.17 d	3.32 ± 0.27 bcd	4.07 ± 0.36 a	12.64 ± 1.14 a	12.19 ± 1.02 a	33.65 ± 1.66 bcde	15.16 ± 0.69 ab
W-MA	20.06 ± 1.87 ab	3.21 ± 0.48 bc	3.87 ± 0.28 a	3.27 ± 0.71 b	9.88 ± 0.34 c	8.26 ± 0.39 d	36.24 ± 1.50 ab	15.22 ± 0.68 ab
Mean	18.89	3.16	3.23	3.24	11.23	10.98	34.69	14.59
Range	(16.42–21.57)	(2.54–3.74)	(2.95–3.87)	(2.16–4.07)	(9.88–12.64)	(8.26–12.55)	(31.13–37.88)	(12.32–16.02)

Values within each column followed by different letters are significantly different at *p* < 0.

**Table 5:** Fatty acid composition, expressed in percentage of total fatty acids, of leaf blades of 12 borage accessions sampled at 150 days after sowing (Mean ± SD (n = 6)).

Acc.	Palmitic C16:0	Palmitoleic C16:1	Stearic C18:0	Oleic C18:1	Linoleic C18:2	GLA C18:3(*n*−6)	ALA C18:3(*n*−3)	Stearidonic C18:04
CV-BA	24.72 ± 2.55 c	2.55 ± 0.49 ab	3.63 ± 0.70 cde	3.82 ± 1.00 bc	10.77 ± 0.85 bc	4.94 ± 0.56 c	36.69 ± 2.05 cd	12.88 ± 0.66 abcd
CV-RO	23.03 ± 1.23 c	2.25 ± 0.47 abc	2.47 ± 0.62 f	3.28 ± 0.70 c	10.53 ± 1.02 bc	5.11 ± 0.41 c	40.14 ± 1.41 ab	13.19 ± 0.94 abcd
CV-RA	23.25 ± 2.08 c	1.72 ± 0.10 cd	3.15 ± 0.67 def	4.85 ± 1.63 ab	11.87 ± 2.25 ab	4.36 ± 1.85 cde	38.64 ± 4.28 abc	12.17 ± 1.96 bcd
CS-CA	31.41 ± 2.29 a	1.95 ± 0.75 bcd	4.65 ± 0.62 ab	5.67 ± 1.10 a	9.42 ± 0.83 cd	2.83 ± 3.28 f	34.99 ± 2.25 de	9.07 ± 1.42 e
BL-RI	23.13 ± 2.05 c	2.24 ± 0.27 abc	3.52 ± 1.06 cde	3.36 ± 1.16 c	9.60 ± 2.49 cd	3.63 ± 1.39 ef	41.59 ± 3.05 a	12.93 ± 0.99 abcd
BL-MO	23.78 ± 1.71 c	2.26 ± 0.82 abc	3.27 ± 0.65 def	3.99 ± 0.73 bc	12.90 ± 1.50 a	6.28 ± 3.00 b	35.93 ± 1.67 cde	11.60 ± 0.86 cd
LR-BU	22.07 ± 2.68 c	2.34 ± 0.53 abc	3.95 ± 1.93 bcd	2.74 ± 0.74 c	10.06 ± 3.67 cd	4.07 ± 2.25 cde	40.85 ± 4.68 ab	13.91 ± 1.19 a
LR-LU	27.36 ± 1.28 b	2.12 ± 0.28 bcd	3.54 ± 0.39 cde	3.05 ± 0.44 c	9.91 ± 1.29 cd	4.76 ± 1.73 cd	37.70 ± 1.46 bcd	11.56 ± 1.36 d
LR-TR	24.53 ± 1.93 c	1.90 ± 0.50 bcd	3.01 ± 0.37 def	3.51 ± 0.93 c	10.66 ± 3.33 bc	4.07 ± 0.90 cde	39.05 ± 2.78 abc	13.28 ± 1.30 abc
W-VI	29.34 ± 1.62 ab	2.95 ± 0.58 a	4.41 ± 0.50 cde	5.32 ± 0.53 a	8.62 ± 3.51 d	3.84 ± 1.13 de	33.26 ± 3.65 e	12.25 ± 1.49 abcd
W-CS	23.38 ± 2.88 c	1.40 ± 0.25 d	2.65 ± 0.52 ef	2.90 ± 1.04 c	13.38 ± 2.96 a	9.57 ± 1.09 a	33.30 ± 3.19 e	13.41 ± 2.81 ab
W-MA	30.10 ± 1.35 a	1.58 ± 0.61 cd	5.07 ± 0.54 a	3.45 ± 0.88 c	9.76 ± 0.70 cd	1.89 ± 0.94 g	38.67 ± 1.98 abc	9.48 ± 1.59 e
Mean	25.56	2.10	3.61	3.85	10.56	4.63	37.58	12.12
Range	(20.06–31.41)	(1.4–2.95)	(2.45–4.65)	(2.74–5.67)	(9.39–13.38)	(1.91–9.57)	(33.26–41.59)	(9.07–13.91)

Values within each column followed by different letters are significantly different at *p* < 0.05.
